# Lipid Transfer Proteins (LTP) with α‑Amylase Inhibitory Activity from *Capsicum chinense* Seeds: Structural and Functional Insights

**DOI:** 10.1021/acsomega.6c00762

**Published:** 2026-06-04

**Authors:** Arielle Pinheiro Bessiatti Fava Oliveira, Larissa Maximiano Resende, Layrana de Azevedo dos Santos, Marciele Souza da Silva, Gabriel Bonan Taveira, Érica de Oliveira Mello, Vitor Batista Pinto, Sarah Rodrigues Ferreira, Antonia Elenir Amancio Oliveira, Fadi Simon de Souza Magalhães, Luis Guilherme Mansor Basso, Andre de Oliveira Carvalho, Rosana Rodrigues, Valdirene Moreira Gomes

**Affiliations:** † Laboratório de Fisiologia e Bioquímica de Microrganismos, Centro de Biociências e Biotecnologia, 28109 Universidade Estadual do Norte Fluminense Darcy Ribeiro, Campos dos Goytacazes, Rio de Janeiro 28013-602, Brazil; ‡ Laboratório de Biologia Celular e Tecidual, Centro de Biociências e Biotecnologia, Universidade Estadual do Norte Fluminense Darcy Ribeiro, Campos dos Goytacazes, Rio de Janeiro 28013-602, Brazil; § Laboratório de Química e Função de Proteínas e Peptídeos, Centro de Biociências e Biotecnologia, Universidade Estadual do Norte Fluminense Darcy Ribeiro, Campos dos Goytacazes, Rio de Janeiro 28013-602, Brazil; ∥ Laboratório de Ciências Físicas, Centro de Ciências e Tecnologias, Universidade Estadual do Norte Fluminense Darcy Ribeiro, Campos dos Goytacazes, Rio de Janeiro 28013-602, Brazil; ⊥ Laboratório de Genética e Melhoramento de Plantas, Centro de Ciências e Tecnologias Agropecuárias, Universidade Estadual do Norte Fluminense Darcy Ribeiro, Campos dos Goytacazes, Rio de Janeiro 28013-602, Brazil

## Abstract

Lipid transfer proteins (LTPs) are small, cysteine-rich antimicrobial peptides widely distributed in plant tissues and involved in various physiological functions, especially defense against pathogens and pests. In *Capsicum chinense*, a species known for its richness in bioactive compounds, LTPs were previously identified in seeds, exhibiting antifungal and α-amylase inhibitory activities. Given this potential, the present study aimed to purify and structurally and functionally characterize the LTP-rich fraction (P10), previously isolated from *C. chinense* seeds. To this end, the P10 fraction was subjected to reversed-phase rechromatography, resulting in three subfractions (R1–R3) with molecular masses close to 9 kDa, which reacted positively with anti-LTP antibodies. The protein identities were confirmed by mass spectrometry, revealing nonspecific LTPs with high similarity to *C. chinense* reference sequences. AlphaFold structural modeling, combined with proteomic mapping, revealed conserved surface-exposed regions, suggesting important functional targets. Furthermore, circular dichroism analyses demonstrated the stability of the secondary structure of LTPs at different temperature ranges, as well as their ability to interact with model lipid membranes. Functional assays confirmed the significant inhibitory activity of these LTPs against *Tenebrio molitor*, human salivary, and swine pancreatic α-amylases, with minimal hemolytic effects. Corroborating these data, molecular docking studies revealed stable interactions between LTPs and target enzymes, with the participation of specific residues at the binding interface, suggesting a possible mechanism of enzyme inhibition. Taken together, the data obtained reinforce the multifunctional nature of LTPs from *C. chinense* seeds and highlight their promising biotechnological potential, especially for applications in agriculture and healthcare.

## Graphical abstract

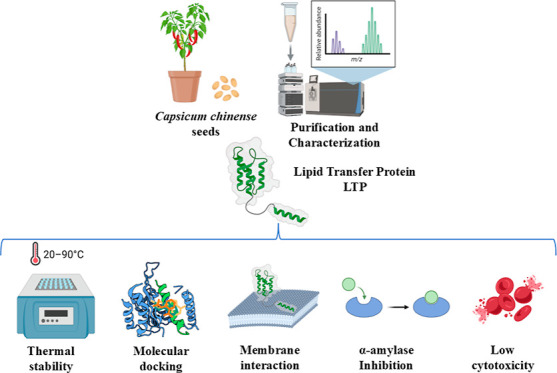

## Introduction

Lipid transfer proteins (LTPs) are small, positively charged proteins widely distributed across plant organs, including seeds, leaves, roots, fruits, and vascular tissues. They are predominantly localized in epidermal cells adjacent to the cuticle but are also detected in embryonic and vascular tissues. Although LTPs are mainly found in the apoplast, their presence has been reported in the cell wall, plasma membrane, and other intracellular compartments, with distribution patterns varying according to environmental conditions and developmental stage.^1,2^

LTPs belong to the antimicrobial peptide (AMP) family and are traditionally classified into two major subclasses, LTP1 and LTP2, based on molecular mass and cysteine content. Advances in phylogenetic and structural analyses have expanded this classification to ten groups (LTP1, LTP2, LTPc, LTPd, LTPe, LTPf, LTPg, LTPh, LTPj, and LTPk), defined by cysteine spacing, intron organization, and polypeptide sequence similarity.^2,3^ Among these, LTP1 proteins—the focus of this study—typically exhibit molecular masses of 9–10 kDa, high isoelectric points (pI 9–10), and a conserved three-dimensional fold composed of four α-helices and one 3_10_ helix stabilized by four disulfide bonds.^4^

LTPs are notable for their multifunctionality and structural stability. Reported biological roles include lipid transport and membrane stabilization, cell wall organization, cuticle and wax formation, signaling processes, and contributions to resistance against biotic and abiotic stresses, seed germination, nodulation, and plant growth. In addition, LTPs may act synergistically with other AMPs.^2,5,6^ Plant defense against pathogens was among the earliest described functions of LTPs, supporting their classification within the Pathogenesis-Related Protein Family 14 (PR14).^7^

Although subclasses such as LTP1 and LTP2 exhibit distinct structural features, the relationship between these subclasses, their isoforms, and functional specialization remains unclear.^8^ The biochemical behavior and biological functions of these variants may differ as a result of structural variations. In this context, LTPs have been described as enzyme inhibitors, supporting the hypothesis that these proteins may act as defense components through enzymatic inhibition.^2,9^ However, although many plant species express LTP isoforms with distinct expression patterns and biological activities, studies directly comparing their enzymatic inhibitory potential remain scarce.

In addition to structural diversity, the ability of LTPs to interact with membranes represents a key feature that may influence their biological activity. Their conserved hydrophobic cavity and amphipathic structure enable binding to a variety of lipid molecules and promote interactions with lipid bilayers. These interactions have been associated with membrane destabilization processes, including increased permeability through lipid removal and electrostatic perturbations mediated by cationic residues present on the protein surface.^10^ Such mechanisms are directly related to the antimicrobial activity of LTPs and their role in plant defense. Furthermore, membrane association may influence protein conformation and the accessibility of molecular targets, potentially affecting interactions with enzymes.

LTPs play an important role in plant defense, acting through different complementary mechanisms, such as direct antimicrobial activity, membrane destabilization, cuticular barrier reinforcement, reinforcement of digestive enzymes in herbivores, and participation in systemic protection pathways.^2,11^ This diversity of functions highlights their relevance in plant immune responses and suggests that their different structural and biophysical properties are related to the variety of biological activities they affect.^11^

Within this context, several studies have demonstrated pronounced antimicrobial activity of LTPs from *Capsicum* species. A comprehensive review compiled antimicrobial peptides, including LTPs isolated from *Capsicum*, highlighting their effectiveness against diverse pathogens.^12^ Subsequent investigations identified additional LTPs in this genus: CaCLTP2 from *Capsicum annuum* leaves showed strong antifungal activity against multiple *Candida* species;^13^ LTP-like fractions from *C. baccatum* and *C. frutescens* seeds were active against *Candida albicans* and *Candida tropicalis*;^14^ and LTP-rich fractions from *Capsicum chinense* seeds exhibited fungistatic effects and induced marked structural alterations in yeast cells.^15^ These findings reinforce the multifunctional nature of *Capsicum* LTPs and underscore their relevance in plant defense mechanisms.

Beyond antimicrobial activity, certain LTPs have been shown to inhibit α-amylases, further expanding their functional scope. In *C. annuum*, Diz et al. first reported inhibition of human salivary α-amylase, suggesting a defensive role against herbivorous insects through impaired carbohydrate digestion.^16^ More recently, in *C. chinense*, a protein fraction (P10) isolated from seeds exhibited immunoreactivity to anti-LTP antibodies and inhibitory activity against *Tenebrio molitor* α-amylase.^15^ Despite the agricultural importance of *C. chinense* and its richness in bioactive compounds, the seed proteome of this species remains relatively underexplored, particularly regarding inhibitors of digestive enzymes.^12^

Based on these considerations, we hypothesize that LTP isoforms present in *C. chinense* seeds exhibit structural and biophysical properties associated with α-amylase inhibition, and that interactions with lipid membranes may influence this inhibitory mechanism. To test this hypothesis, the present study aimed to further characterize the LTP-rich fraction previously identified in *C. chinense* seeds through purification and structural analysis, combined with an expanded functional evaluation of its inhibitory activity against different α-amylases. Additionally, secondary structure, interactions with model lipid membranes, molecular docking predictions, and hemolytic activity were investigated. This integrated approach provides new insights into the relationship between LTP structure, membrane interaction, and enzyme inhibition, highlighting their potential role in plant defense and their promising biotechnological applications.

## Results

### Rechromatography of Fraction P10 and Characterization of Subfractions R1, R2, and R3

Given that fraction P10 had been previously isolated by reversed-phase chromatography, we performed a new purification step by HPLC rechromatography to improve resolution. The chromatographic profile (Figure 1A) resolved three main subfractions R1, R2, and R3. Tricine–SDS–PAGE of these subfractions (Figure 1B) showed protein bands with apparent molecular masses of ∼6.5 to 14.2 kDa. The repeated banding pattern across R1, R2 and R3 reinforces the efficiency of the fractionation and indicates structural similarities among the major components. Because P10 had been characterized previously as containing an LTP peptide of ∼9 kDa, we performed immunodetection by Western blotting on the subfractions. Immunoreactive bands were detected in R1, R2, and R3, as well as in the original P10 fraction (Figure 1C), indicating the presence of the peptide of interest in all samples analyzed.

**Figure 1:**
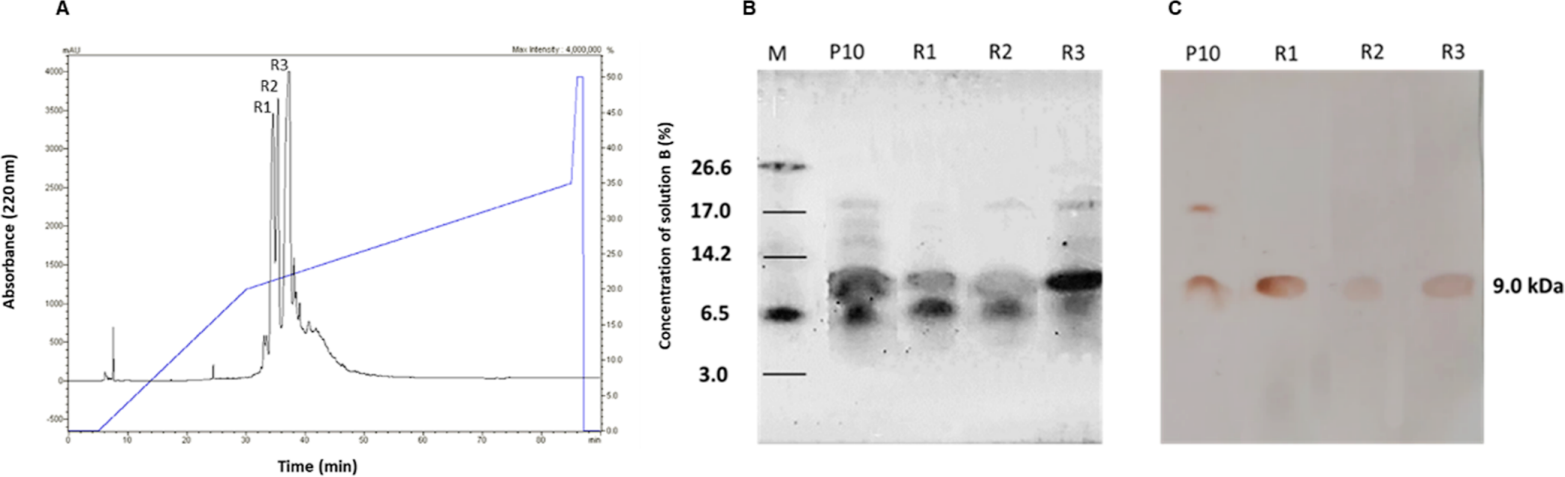
(A) Rechromatography of the P10 fraction obtained by reversed-phase chromatography on a μRP C18 column. The column was equilibrated with 0.1% TFA (Solvent A) and elution was performed using a linear propanol gradient (Solvent B) at a flow rate of 0.5 mL·min^–1^. (B) Tricine-SDS-PAGE analysis of fractions obtained from rechromatography. *M*: molecular mass marker (kDa); P10: fraction obtained by HPLC; R1, R2, and R3: rechromatographed subfractions. (C) Western blot analysis of P10 and its subfractions using anti-LTP antibodies.

### Characterization of the Subfractions by Mass Spectrometry

To identify the peptide fragments present in R1, R2, and R3, we analyzed the protein bands highlighted in red in Figure 1B by mass spectrometry. Analyses employed Mascot and PLGS, and the resulting sequences were queried against NCBI and UniProt/BLASTp. For fragment comparison, we used two reference sequences: A0A2G3DEZ6_CAPCH (nonspecific lipid-transfer protein B, *C. chinense*; GenBank: PHU29557.1) and A0A2G3DEZ3_CAPCH (nonspecific lipid-transfer protein 1, *C. chinense*; GenBank: PHU29554.1). The matched peptides showed 100% identity to the reference sequences. Sequence alignment across the subfractions indicated identities of 80 to100% and physicochemical similarities of 90 to 100%. Coverage indices were: R1—A0A2G3DEZ6 (23.3%) and A0A2G3DEZ3 (14.2%); R2—A0A2G3DEZ6 (32.5%) and A0A2G3DEZ3 (14.4%); R3—A0A2G3DEZ6 (10.8%) and A0A2G3DEZ3 (18.1%) (Figure 2A–C). Conserved regions are colored; insertions are shown as dashes (−).

**Figure 2:**
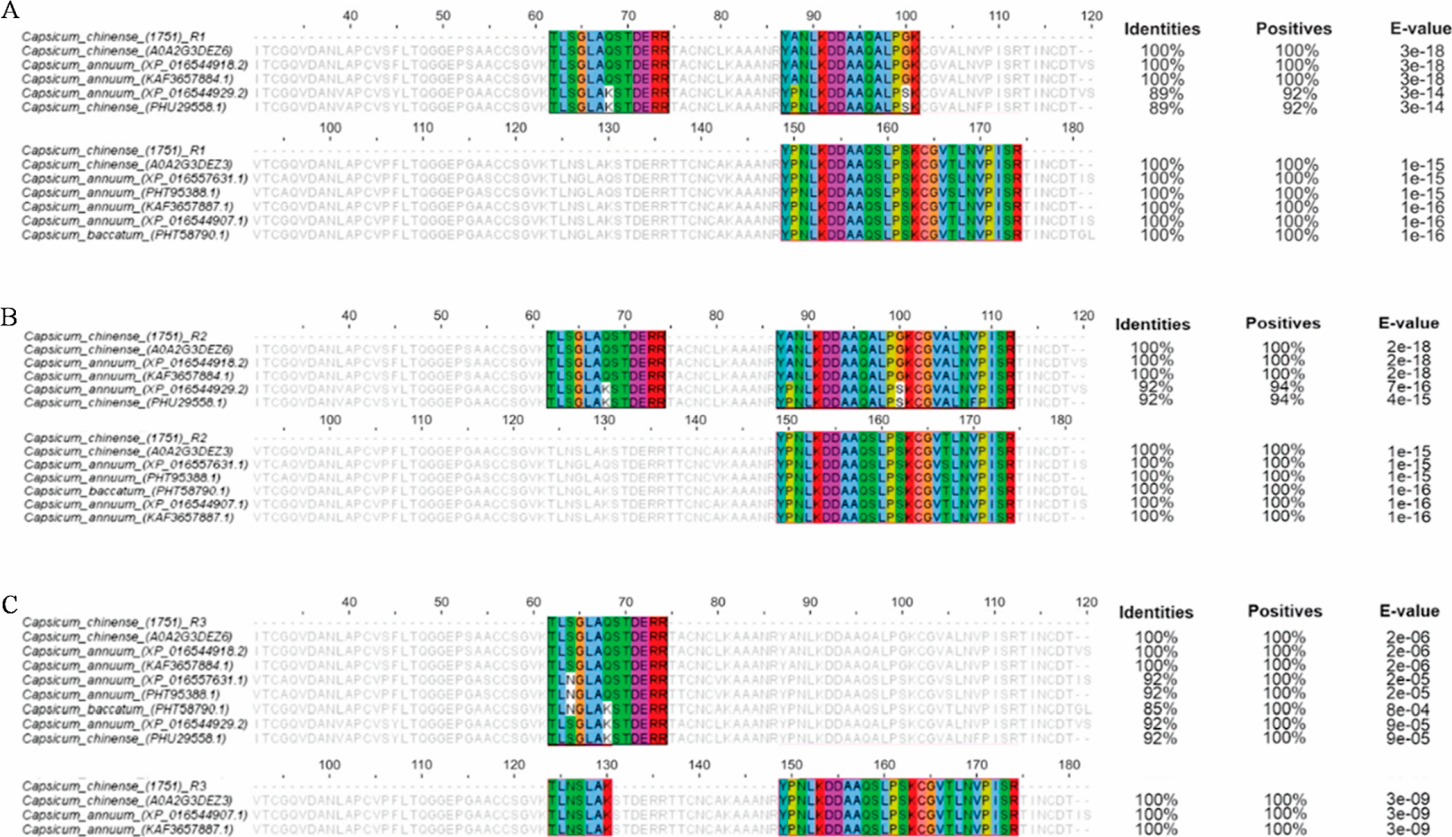
Sequence alignment of peptide fragments identified in fractions R1 (A), R2 (B), and R3 (C) obtained from *Capsicum chinense* seeds (UENF accession 1751). Homologous sequences were identified using BLAST, aligned with Clustal Omega, and visualized in Jalview. Identity values represent the proportion of identical amino acid residues and are highlighted. Conserved substitutions (positives), defined as residues with similar physicochemical properties, are indicated in gray. Gaps (−) were introduced to optimize sequence alignment.

### Structural Modeling and Mapping of Peptide Fragments Identified in *C. chinense* LTPs

To verify the structural localization of the fragments obtained by mass spectrometry, three-dimensional modeling with the A0A2G3DEZ3_CAPCH and A0A2G3DEZ6_CAPCH proteins was performed using the AlphaFold platform. The predicted structures were visualized using PyMOL, highlighting the peptide fragments identified in subfractions R1, R2, and R3.

Figure 3A shows the fragments aligned with the A0A2G3DEZ3 protein. This demonstrates that the structural regions are distributed between α-helices and external loops. The results showed particular emphasis on the peptides TLNSLAK, DDAAQSLPSK, CGVTLNVPISR, and YPNLKDDAAQSLPSK. Figure 3B shows that the fragments mapped to A0A2G3DEZ6 also exhibited conformations consistent with the surface regions. These included the peptides YANLKDDAAQALPGK, CGVALNVPISR, and TLSGLAQSTDERR. These fragments were positioned in the external regions of the protein structure, indicating possible exposure to interactions with other biomolecules. The AlphaFold models provided high structural confidence (shown in blue), indicating the high reliability of the generated structural models and enabling accurate functional inferences.

**Figure 3:**
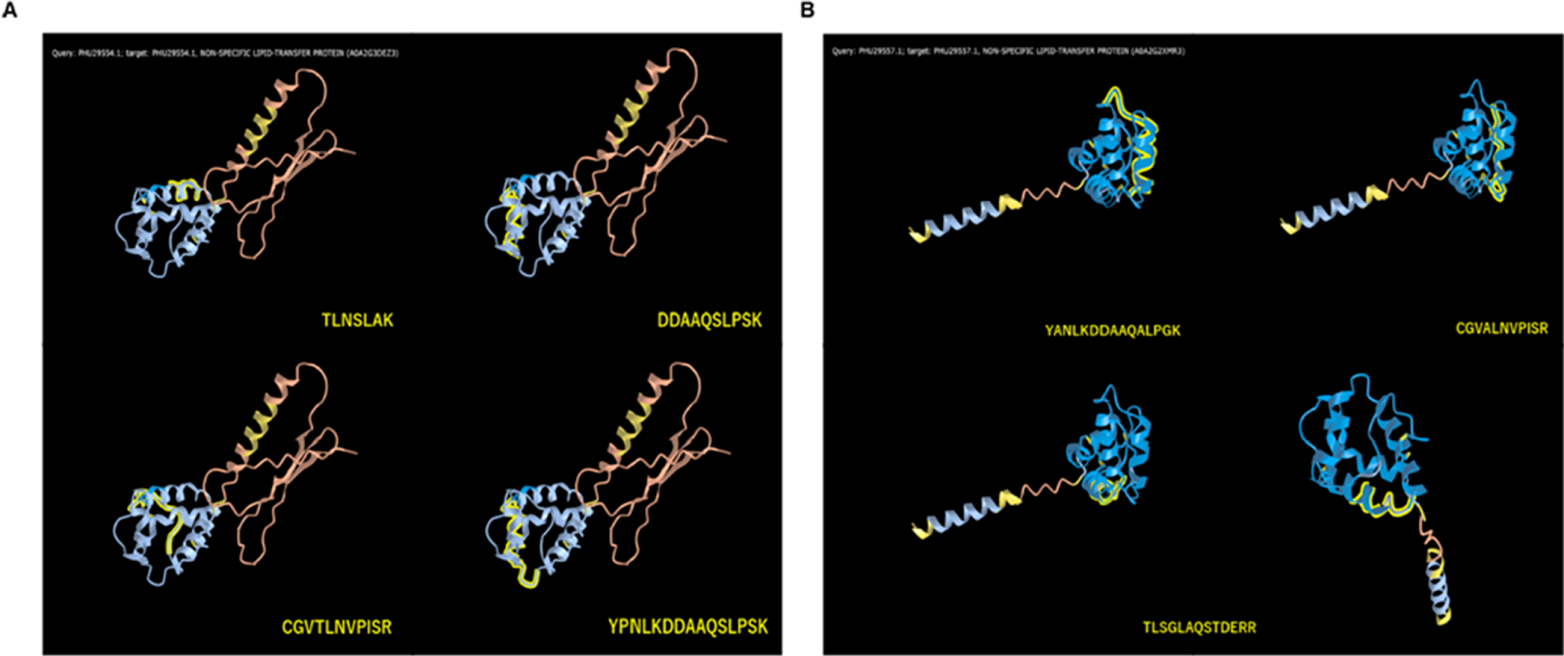
Predicted three-dimensional structures of *Capsicum chinense* LTP proteins highlighting the peptide fragments identified in the subfractions. (A) Fragments aligned with protein A0A2G3DEZ3_CAPCH (nonspecific lipid transfer protein 1; GenBank: Phu29554.1). (B) Fragments aligned with protein A0A2G3DEZ6_CAPCH (nonspecific lipid transfer protein; GenBank: Phu29557.1). Structures were generated using AlphaFold and visualized in PyMOL. Identified peptide fragments are highlighted in yellow. Model confidence is indicated by the pLDDT score: dark blue (>90, very high confidence), light blue (70–90, confident), yellow (50–70, low confidence), and orange (<50, very low confidence).

### Effects of Interactions with Membrane Models on the Secondary Structure of Proteins R1, R2 and R3

Figure 4 shows the far-UV CD spectra of R1, R2, and R3 in the presence and absence of SUVs composed of different lipid mixtures. Panels A, B, and C show that all proteins exhibited characteristic double minima at 208 and 222 nm in aqueous solution, indicating a predominantly α-helical conformation. This helical structure was relatively thermally stable, as evidenced by (i) the persistence of α-helical spectral features even at 90 °C, indicating only partial unfolding (see Figure 5C) and (ii) a gradual decrease in ellipticity at 222 nm as the temperature increased, with no clear cooperative transition to a fully denatured state (see Figure 5D). Notably, partial refolding was observed only for R1 and R3 after cooling back to 20 °C (Figure 5A,C).

**Figure 4:**
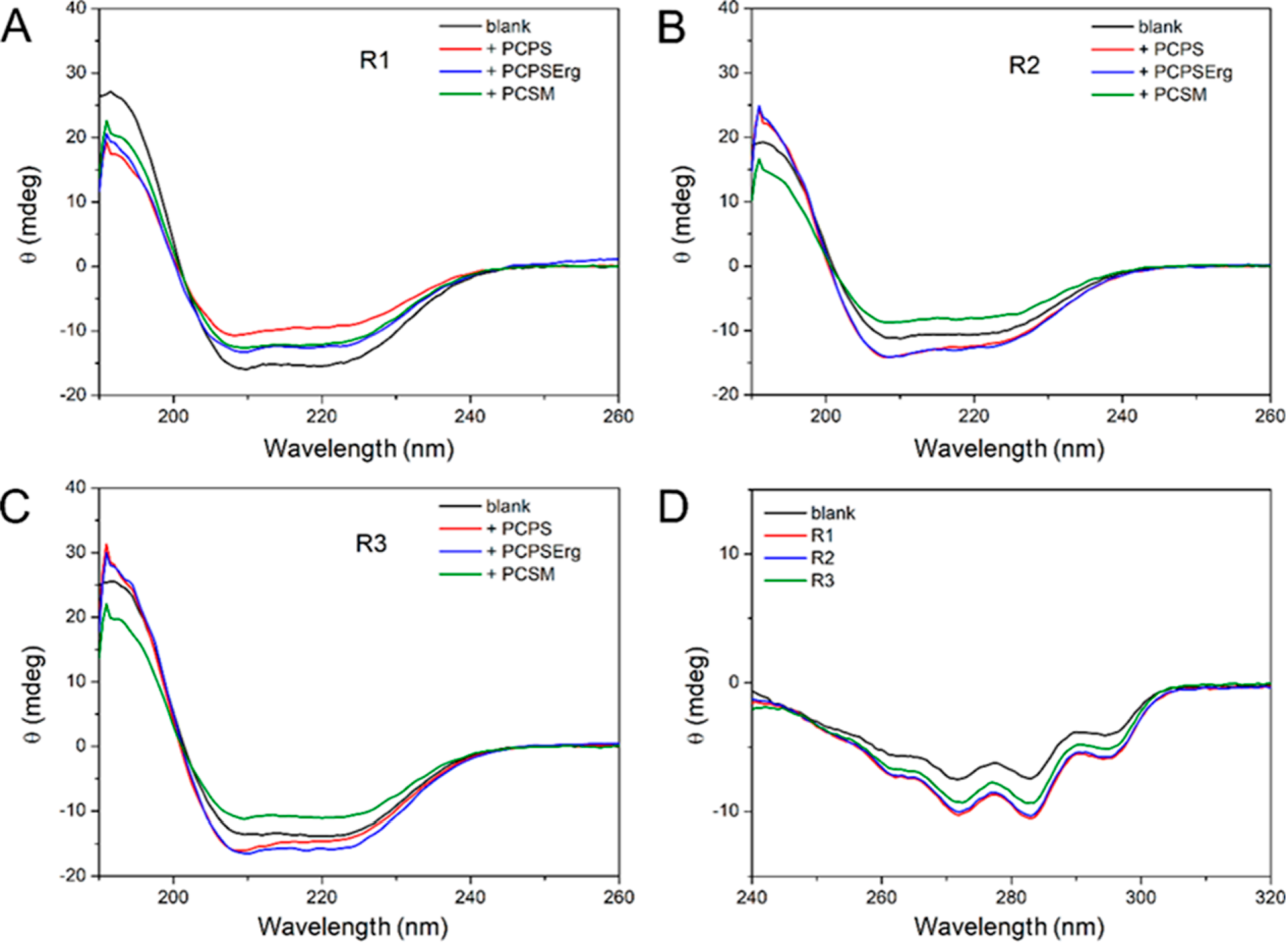
Far-UV circular dichroism (CD) spectra of 30 μM (A) R1, (B) R2, and (C) R3 in the absence (black) and presence of 1 mM small unilamellar vesicles (SUVs) composed of POPC/POPS (red), POPC/POPS/ergosterol (blue), and POPC/sphingomyelin (green). (D) Near-UV CD spectra of ergosterol-containing samples in the absence (black) and presence of the proteins. Spectra were recorded at 37 °C and are presented in millidegrees.

**Figure 5:**
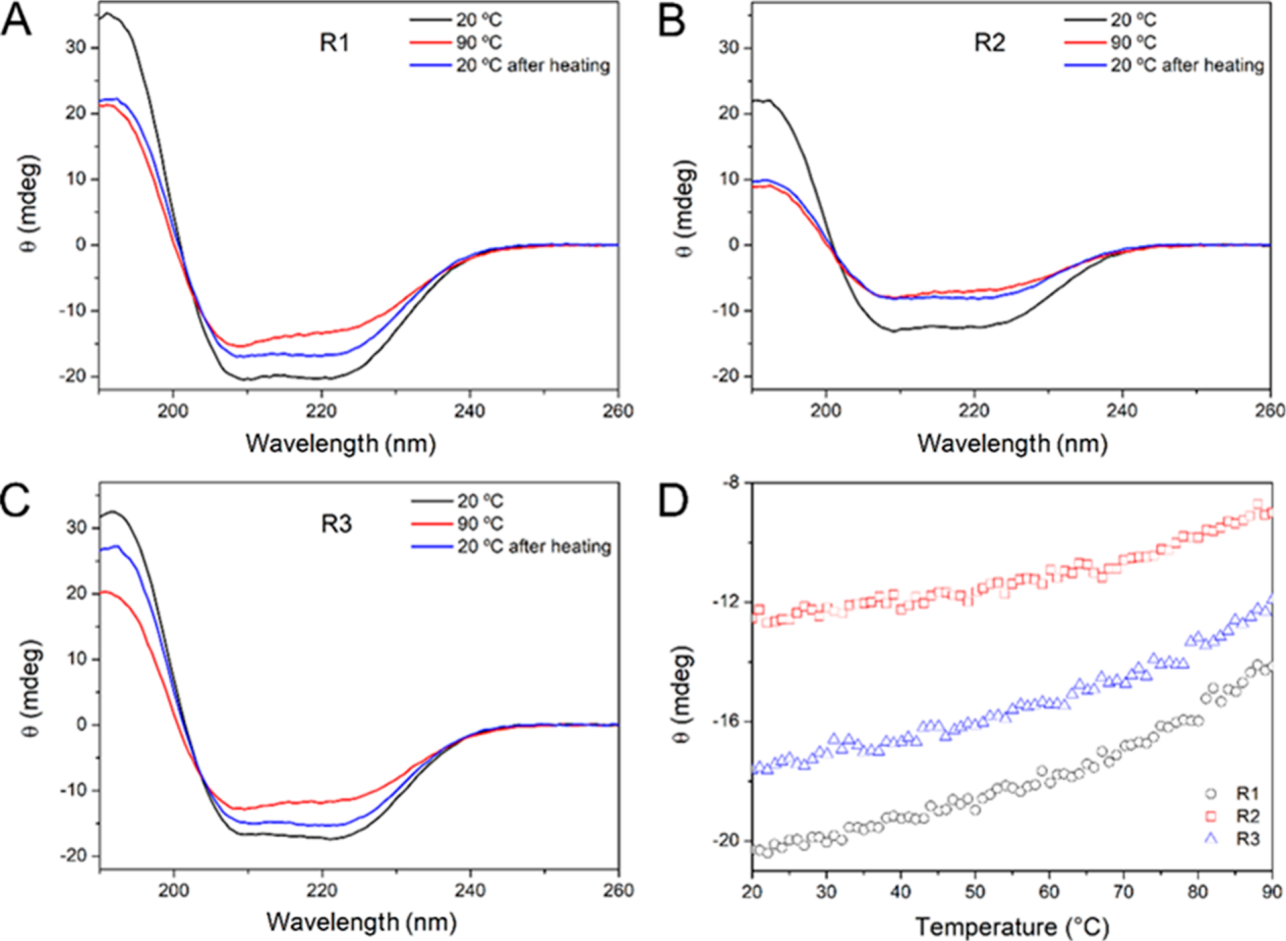
Thermal denaturation of R1, R2, and R3 in aqueous solution. (A–C) Far-UV CD spectra of 30 μM (A) R1, (B) R2, and (C) R3 recorded at 20 °C, 90 °C, and after cooling back to 20 °C. (D) Ellipticity at 222 nm monitored from 20 to 90 °C. Spectra were recorded in ultrapure water and are expressed in millidegrees.

After the addition of SUVs, distinct spectral changes were observed for each protein, reflecting different structural responses to membrane interactions. For R1, all membrane models induced a reduction in CD signal intensity, indicating a decrease in α-helical content. Interestingly, R1 retained a more helical structure in the ergosterol-containing fungal membrane (POPC/POPS/Erg) than in the POPC/POPS model without ergosterol. The R1 spectra in the POPC/POPS/Erg and POPC/SM mammalian-like membranes were similar in terms of helical content. This behavior suggests that membrane order and packing may play a role in modulating the conformation of R1. Conversely, R2 and R3 exhibited increased helicity upon interaction with POPS-containing anionic membranes but reduced helical content in the POPC/SM membrane. Thus, the membrane surface charge plays a key role in modulating the secondary structure of R2 and R3.

In the near-UV region (240–350 nm; Figure 4D), three distinct negative bands were exclusively observed in the ergosterol-containing membranes at approximately 272, 283, and 294 nm. These signals corresponded to the electronic transitions of ergosterol .^23,24^ Their intensities changed upon addition of the proteins, suggesting that all three subfractions altered the local ergosterol environment within the membrane. R1 and R2 produced the most pronounced effects, as evidenced by stronger negative bands compared to R3 (Figure 4D). These results suggest that protein binding induces changes in the conformational and/or orientational state of ergosterol in the lipid bilayer, consistent with membrane-context-dependent interactions.

### Inhibitory Activity of Fractions on Different α-Amylases

We quantified inhibition by fractions P10, R1, R2, and R3 against *T. molitor*, porcine pancreatic, and human salivary α-amylases (Figure 6). For *T. molitor* α-amylase, inhibition was 98% (P10), 93% (R3), 72% (R2), and 55% (R1); differences among fractions were statistically significant (*p* < 0.0001) (Figure 6A). For porcine pancreatic α-amylase, inhibition was 89% (P10), 84% (R1), 81% (R2), and 80% (R3) (Figure 6B). For human salivary α-amylase, inhibition was 93% (P10), 91% (R1), 85% (R2), and 92% (R3) (Figure 6C). Control conditions (EDTA and BSA) are shown in Figure 6.

**Figure 6:**
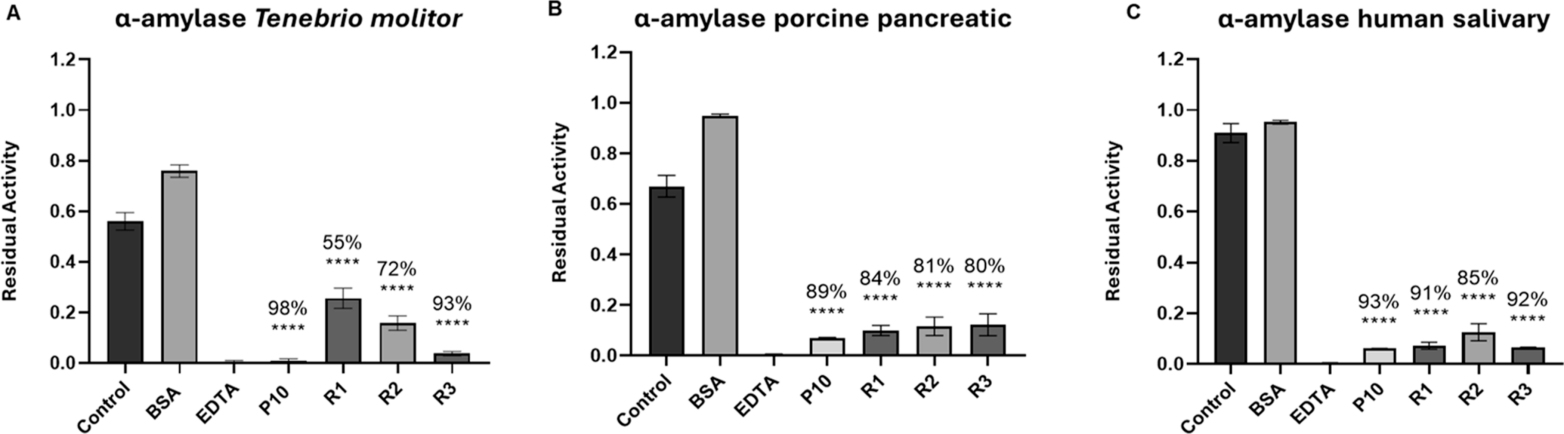
Inhibitory effects of fractions rechromatographed in reversed phase (R1, R2 and R3-50 μg/mL) on the activity of α-amylases from *Tenebrio molitor* (A), porcine pancreatic (B) and human salivary (C). Control—α-amylase; EDTA—positive control 5 mM. BSA—negative control 10 μg. (%)—Percentage of enzymatic activity caused by the fractions. (****) Asterisks indicate significant difference (*p* < 0.0001) by Dunnett’s test.

### Molecular Docking Analysis between LTP Proteins and α-Amylase

The α-amylase model was obtained from Protein Data Bank (PDB ID: 1JAE) and highlights the center of the catalytic site composed of residues D185, E222, and D287 in yellow (Figure 7A). In both LTP−α-amylase complexes, the LTPs were found to be positioned in the catalytic cleft of the enzyme, potentially obstructing access to the substrate. The calculated binding free energies for the LTPB-α-amylase and LTP1-α-amylase complexes were −15.7 kcal/mol and −16.8 kcal/mol, respectively, indicating spontaneous and energetically favorable interactions (Figure 7B). The LTP residues that came into contact with α-amylase are highlighted in red in Figure 7C.

**Figure 7:**
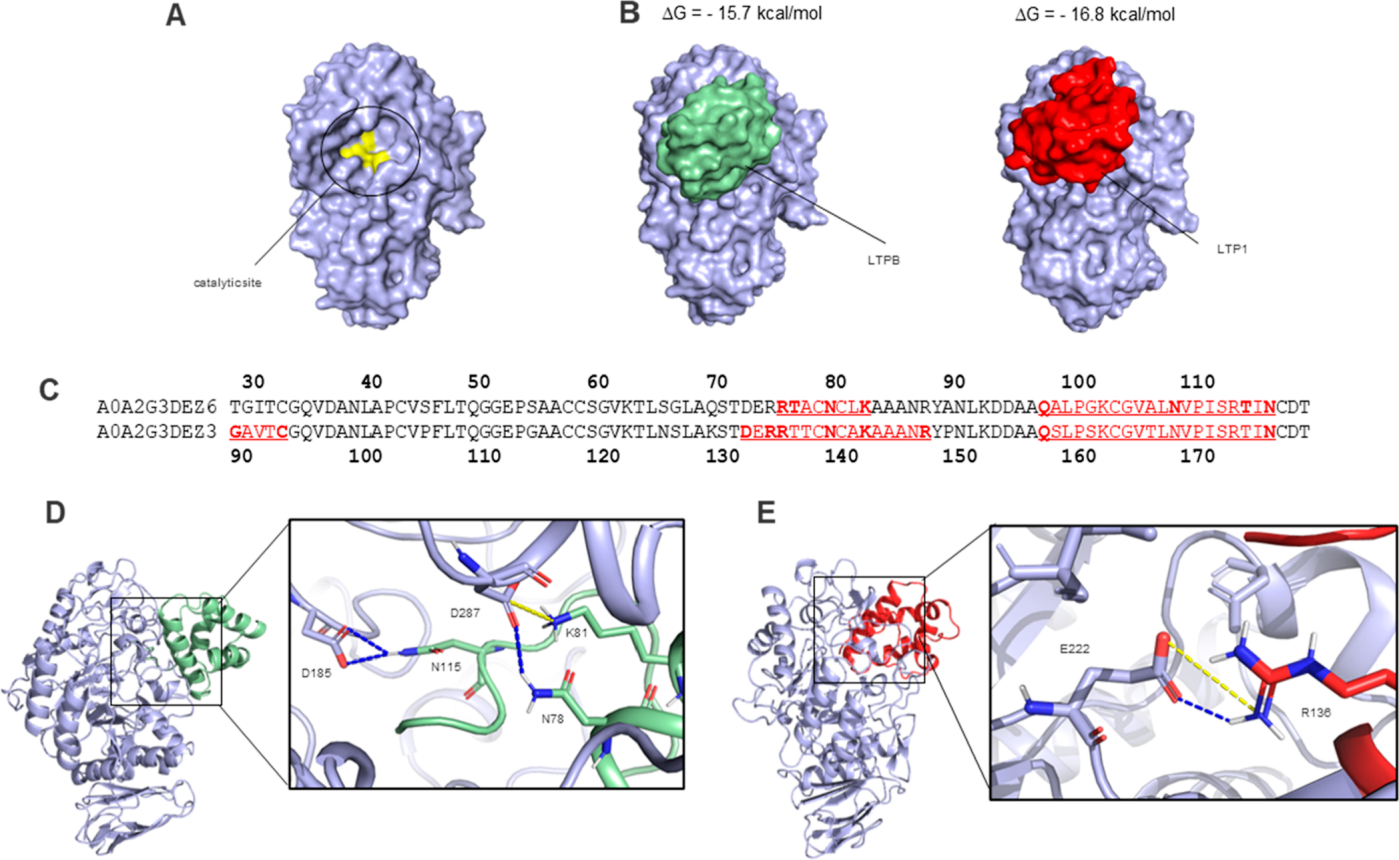
(A) The structural model of *Tenebrio molitor* larval α-amylase (PDB: 1JAE) represented in surface model where in yellow is represented the catalytic site. (B) Alpha-amylase-LTP complex obtained by protein–protein docking studies suggesting obstruction of carbohydrate binding cleft. (C) Primary structure of LTP with the region of amino acid residues that make contacts with alpha-amylase highlighted in red and amino acids directly involved in the interaction in bold. (D) LTP-Alpha-amylase interaction where amino acid residues from active site of alpha-amylase D185 and D287 are interacting with amino acid residues from LTP (A0A2G3DEZ6). (E) LTP-Alpha-amylase interaction where amino acid residues from active site of alpha-amylase E222 is interacting with amino acid residues from LTP (A0A2G3DEZ3). Hydrogen bonds are showed in blue dashes. Salt bridges are showed in yellow dashes.

Docking analyses indicated that both LTPs interacted with the enzyme through multiple hydrogen bonds, salt bridges, and hydrophobic interactions. Representative interactions included the catalytic residue D185 of α-amylase forming a hydrogen bond with N115 of LTPB, and residue D287 forming a hydrogen bond with N78 and a salt bridge with K81 (Figure 7D). In the LTP1-α-amylase complex, E222 interacts with R136 of LTP1 through both a hydrogen bond and a salt bridge (Figure 7E).

### Hemolytic Activity of Fraction P10

We evaluated hemolytic activity of fraction P10 at 300, 150, 75, and 35.5 μM. The positive control (Triton X-100) showed mean hemolysis above 100%, whereas all P10 concentrations were lower (*p* < 0.05) and approached the negative control (PBS). Only the Triton X-100 group differed significantly from the other groups (*p* < 0.05).

## Discussion

Rechromatography of fraction P10 separated three main components (R1, R2, R3) that, despite prior purity, revealed peptides with similar structures and distinct characteristics. Immunoblotting and mass spectrometry associated these components with *C. chinense* LTPs. Building upon these results, the recurring protein banding pattern among R1, R2, and R3 suggested isoforms, a phenomenon described in different plant species. In *Lens culinaris*, a subfamily of eight isoforms (Lc-LTP1-Lc-LTP8) has been identified.^17^ Similarly, three isoforms with distinct expression profiles have been identified in *Pisum sativum*: Ps-LTP1, Ps-LTP2, and Ps-LTP3.^18^ In *Capsicum*, Diz et al. reported three Ca-LTP1 isoforms purified from *C. annuum* seeds with masses close to 9 kDa but different isoelectric points (pI 6.0, 8.5, and 9.5), indicating subtle structural variations within species.^19^ In this context, the separation of P10 into R1, R2 and R3 may indicate isoforms of the same LTP with slight structural differences, supported by variations in amino acid fragments and similarities among electrophoretic profiles and physicochemical properties. Potential origins include alternative splicing, post-translational modifications, or expression of paralogous genes; as observed in *P. sativum*, *L. culinaris*, and *C. annuum*, such variation may reflect specialized functions related to germination, lipid transport, and stress responses.

However, although the isoform hypothesis is supported by combined electrophoresis, Western blotting, and proteomics data, alternatives for the observed heterogeneity must be considered. Post-translational modifications (PTMs), common in plant LTPs, can generate distinct proteoforms from the same polypeptide chain, leading to differences in electrophoretic mobility, mass spectrometry profiles, and antibody recognition.^20–22^ Furthermore, partial proteolysis during extraction and purification steps can produce peptide fragments with altered migration patterns, contributing to the observed diversity among the fractions.^20,21^ Another possibility is the presence of closely related parallel proteins, derived from gene duplication events, which may exhibit high sequence similarity while maintaining subtle structural differences detectable by sensitive techniques such as mass spectrometry.^20^ Therefore, although current data consistently indicate that R1, R2, and R3 belong to the LTP family, the classification of these fractions as distinct isoforms should be further investigated. Further analyses, including detailed characterization of post-translational modifications (PTMs) and optimization of sample preparation to minimize proteolysis, will be essential to conclusively distinguish between true isoforms and other structurally related variants.^23,24^

To further validate the identity of these isoforms, immunodetection by Western blotting using LTP-specific antibodies confirmed peptides of interest with mass close to 9 kDa in the original fraction and in all subfractions (Figure 1C), indicating that the recognized epitope remained intact after successive purification steps. This behavior has been reported for natural LTPs.^14,15,19^ Despite structural variations among R1, R2 and R3, the fractions shared conserved immunogenic regions, a typical characteristic of the LTP family that is possibly associated with residues involved in lipid interactions and biological functions.^24^ Consistent with this immunological evidence, mass spectrometry identified fragments showing high similarity to *C. chinense* LTP proteins in the A0A2G3DEZ6 and A0A2G3DEZ3 databases (Figure 2). Partial but consistent coverage and conservation of specific sequences, such as DDAAQSLPSK, CGVALNVPISR, and TLSGLAQSTDERR, suggest that the isolated peptides belong to the same functional family. These indices are comparable to those reported previously, where smaller, highly conserved fragments were sufficient to attribute functional identity.^13–15^

Structural modeling provided further support for this interpretation. AlphaFold predicted structures were visualized using PyMOL (Figure 3) and indicated that the detected fragments were located in external regions of the proteins, including exposed loops and helices. Thus, these regions may be involved in interactions with ligands or receptors. This corroborates the hypothesis that these LTPs may function in plant signaling or defense processes, consistent with findings reported for *Capsicum* LTPs.^14^

In agreement with these predictions, the CD spectra (Figure 4) showed secondary structure consistent with typical LTP1 characteristics. The LTP1 conformation is predominantly α-helical around a hydrophobic core stabilized by disulfide bonds, conferring high thermal stability and strength and the ability to interact with lipids through a hydrophobic internal cavity, as observed for other plant LTPs.^25^ The thermal stability of the subfractions (Figure 5) reinforces this behavior; partial refolding after cooling in R1 and R3 indicates resilient structural elements important for maintaining biological activity. Moreover, the conformational sensitivity to SUVs highlighted responsiveness to the lipid microenvironment, which is expected for proteins involved in lipid recognition or transfer.^2^ Studies have shown that phosphatidylserine (PS) and ergosterol in fungal model membranes lead to greater bilayer compaction and ordering, affecting protein–surface interactions.^26–28^ In our data, membrane lipid composition directly influenced subfraction structural stability. Specifically, R1 displayed a significant reduction in helical content across lipid models, particularly in POPC/POPS (PCPS) and POPC/SM (PCSM), suggesting pronounced structural reorganization. In contrast, R2 and R3 exhibited increased helicity in the PCPS membrane. The presence of sphingomyelin (PCSM model), which resembles a mammalian membrane, resulted in a slight loss of helical content, which was more evident for R2. This finding reinforces the idea that the chemical nature and charge of the lipid bilayer modulates the conformational stability of these proteins.

Further evidence came from near-UV CD spectra (Figure 4D), where bands associated with ergosterol (a typical fungal membrane lipid) were detected, reflecting changes in the local environment of the sterol within the bilayer. These bands appeared only in the presence of ergosterol and their intensities changed upon addition of the proteins, particularly R1 and R2. This indicates that these LTPs perturb the local ergosterol environment, likely altering its conformational and/or orientational state within the membrane. This behavior is consistent with previous reports on plant antimicrobial peptides.^29^ In our study, the CD spectra revealed that R1 and R2 also underwent pronounced structural changes in the presence of ergosterol-containing membranes, whereas R3 was less affected, suggesting isoform-specific interactions. These observations indicate that the lipid composition of fungal-like membranes modulates protein structure and may influence their functional properties. Thus, the studied subfractions exhibited structural plasticity dependent on the lipid environment, a characteristic consistent with bioactive plant defense proteins and potentially relevant to their antimicrobial function.

Beyond their structural plasticity and membrane interactions, LTPs can act as natural inhibitors of the α-amylase enzyme, thereby expanding their functional and biotechnological scope. Zottich et al. isolated *Cc*-LTP1 from coffee (*Coffea canephora*), which was shown to inhibit mammalian α-amylase in vitro.^30^ Meanwhile, Vu-LTP1.1 from beans (*Vigna unguiculata*) was found to reduce human salivary α-amylase activity by approximately 78% at 20 μM through a mechanism involving direct interaction with positively charged residues such as Arg_39_.^31^ In the present study, P10 demonstrated inhibitory activity, achieving 98% inhibition of *T. molitor* α-amylase (Figure 6A). After chromatographic separation, the subfractions exhibited potent inhibitory activity ranging from 55% to 93% against the insect enzyme and exceeding 80% against porcine pancreatic and human salivary α-amylases. The reduction observed after fractionation may be attributable to the dissociation of isoforms or complementary structures present in the original fraction, whose combined effect may favor more efficient interactions with enzyme active sites. It is important to emphasize that the inhibition assays were performed at a single concentration and quantitative parameters were not determined due to material limitations. Further kinetic studies are part of ongoing work.

These inhibitory effects carry both ecological and biomedical implications. Ecologically, inhibition of α-amylases in insects such as *T. molitor* supports the idea that LTPs act as plant defense components by interfering with the digestion of polysaccharides by herbivores, as previously demonstrated for defensins and LTPs of plant origin.^31–33^ Studies have shown that Vu-LTP1.1 can effectively inhibit both human salivary and intestinal α-amylase from the insect *Callosobruchus maculatus*, thereby confirming its role as a natural defense agent.^32^ From the physiological and clinical perspectives, the inhibition of human α-amylases has been investigated as a complementary approach to postprandial glycemic control, which is relevant for managing type 2 diabetes.^34^ Furthermore, hyperactivity of pancreatic α-amylase has been linked to the pathophysiology of acute pancreatitis, an inflammatory condition involving the excessive release of digestive enzymes. In this context, the LTPs characterized here may represent promising candidates for future investigation in development of nutraceuticals, functional foods, and bioactive compounds with potential applications in metabolic health and sustainable agriculture owing to their strong inhibitory effect and thermal stability. However, these perspectives are based on in vitro enzymatic assays, and further studies, including vivo models, are required to validate their potential biological effects.

Considering the substantial inhibition observed against *T. molitor* α-amylase, a molecular docking study was conducted to explore the potential mechanisms of the interaction between LTPs and the enzyme (Figure 7). The simulated complexes revealed favorable binding energies ranging from −15 to −17 kcal/mol, suggesting stable interactions with the regions in proximity to key catalytic residues (D185, E222, and D287). These interactions involved hydrogen bonds and electrostatic contacts, indicating that LTPs may associate with the active site or nearby regions of the enzyme (Figure 7). Although these findings are consistent, it is important to emphasize that docking predictions alone do not provide direct evidence of the inhibitory mechanism. Instead, they offer structural hypotheses that may help explain the experimental inhibition observed. This interaction pattern previously described mechanisms for Vu-LTP1.1 and *C. annuum* peptides.^19,31^ Similar studies on plant defensins have indicated comparable interactions with insect digestive enzymes, reinforcing the hypothesis of functional convergence among various AMP classes.^33^ Therefore, the docking results should be interpreted as supportive of possible binding modes rather than definitive proof of enzyme inhibition mechanisms. Taken together with the experimental data, these findings reinforce the potential of LTPs as enzyme-interacting molecules, while highlighting the need for further studies to elucidate their precise mode of action. This architecture achieves multiple biological functions. The surface loops of these proteins, which are often composed of basic or hydrophilic residues, constitute the key regions for enzyme recognition. These loops function as ligands that mimic substrates or occupy catalytic sites. An important aspect to consider is the relationship between membrane interaction and α-amylase inhibition. CD data show that the conformation of the LTP subfractions is modulated by membrane composition, indicating structural plasticity in response to the lipid environment. In parallel, enzymatic assays and docking analyses demonstrate that these proteins interact with α-amylase and inhibit its activity. It is therefore plausible that membrane-induced conformational changes influence the exposure and orientation of residues involved in enzyme recognition. Thus, membrane interaction and enzyme inhibition may represent interconnected aspects of protein function, although further studies are required to define their mechanistic coupling.

Finally, to assess the potential toxicity of these proteins, hemolytic activity was evaluated.

The hemolytic activity of the P10 fraction was assessed using sheep red blood cells at concentrations ranging from 35.5 to 300 μg·mL^–1^. No significant hemolysis was observed at any of the tested concentrations, indicating low cytotoxicity toward mammalian erythrocyte membranes. These results are consistent with the behavior reported for other plant LTPs, which typically exhibit selective activity toward microbial membranes while preserving mammalian cell integrity. The absence of hemolytic effects under the tested conditions supports the potential applicability of these proteins in biotechnological and biomedical contexts, although further studies are required to confirm their safety in vivo. The hemolysis assay is widely used to screen antimicrobial agents that target plasma membranes.^32^ The behavior observed here is consistent with other plant LTPs described in the literature. Cherene et al. demonstrated that CaCLTP2 exhibited only a weak hemolytic effect at 200 μg/mL and presented a CC_50_ value > 400 μg/mL.^13^

## Conclusion

In this study, we demonstrated that the P10 fraction of *C. chinense* contains LTP isoforms (R1, R2 and R3) that are structurally similar but functionally distinct, and that these isoforms exhibit high α-amylase inhibition activity against different substrates. The isolated subfractions maintained their structural stability and selective interactions with membrane lipids, and P10 exhibited low hemolytic toxicity, thereby reinforcing the potential of these LTPs as multifunctional bioactive peptides. Together with the molecular docking evidence, the data obtained in this study support the role of these LTPs as natural inhibitors with applicability in agricultural biotechnology and healthcare.

## Methods

### Extraction of Peptides from *Capsicum chinense* Seeds

Seeds of the UENF 1751 accession of *C. chinense* Jacq. were provided by the Laboratório de Melhoramento Genético Vegetal, Centro de Ciências e Tecnologias Agropecuárias, Universidade Estadual do Norte Fluminense Darcy Ribeiro (UENF), Campos dos Goytacazes, Rio de Janeiro, Brazil.

Peptide extraction and partial purification were performed as previously described.^15^ Briefly, seeds were macerated with liquid nitrogen and homogenized in phosphate buffer (0.01 M Na_2_HPO_4_, 0.015 M NaH_2_PO_4_, 0.1 M KCl, 1.5% EDTA; pH 5.4) at a 1:10 (m/v) ratio under continuous stirring for 2 h at 4 °C. The crude extract was centrifuged at 7830*g* for 45 min at 4 °C and filtered, followed by ammonium sulfate precipitation (0–70% saturation) for 16 h at 4 °C.

The protein precipitate was resuspended in distilled water, heat-treated at 80 °C for 15 min, centrifuged at 7830*g* for 45 min at 4 °C, and dialyzed against distilled water for 72 h. The resulting lyophilized material was designated as the seed protein extract (SPE).

### Purification and Obtaining of the P10 Fraction

The seed protein extract (SPE) was fractionated by anion-exchange chromatography on a DEAE-Sepharose column equilibrated with 100 mM Tris–HCl buffer (pH 8.0). Elution was performed using the same buffer containing 1 M NaCl, with absorbance monitored at 280 nm, yielding two fractions, designated C1 and C2.

Fraction C1 was further separated by reversed-phase high-performance liquid chromatography (RP-HPLC) on a C18 column using a linear gradient of propanol in 8.77 mM trifluoroacetic acid (TFA) as the mobile phase. Elution was monitored at 220 nm using a diode-array detector at a flow rate of 0.5 mL/min and a column temperature of 40 °C. The resulting subfractions were lyophilized, and one of them, designated P10, was subjected to RP rechromatography to improve resolution.

Fraction P10 was purified by RP-HPLC using a HIBAR LiChrosorb RP-18 column (25 cm × 4.6 mm, 5 μm; Merck) coupled to a C18 precolumn (Pelliguard LC-18, 2 cm; Supelco). The sample was solubilized in ultrapure water (∼1 mg/mL), and a volume of 200 μL was injected. Separation was performed at a flow rate of 500 μL/min and a temperature of 40 °C. The chromatographic program started with 100% solvent A (8.77 mM TFA in water) for 15 min, followed by a linear increase of solvent B (propanol containing 8.77 mM TFA) from 0% to 50% between 15 and 115 min. Solvent B was then reduced to 0% over 1 min and maintained until 120 min. Elution was monitored at 220 nm using a diode-array detector.

### Protein Content Quantification

Protein concentration was determined using the bicinchoninic acid (BCA) assay, as described by Smith et al. with minor adaptations for this study.^35^ Bovine serum albumin (BSA; Sigma-Aldrich) was used as the standard. Samples and standards were distributed in 96-well microplates and incubated with the BCA reagent according to the manufacturer’s instructions. Absorbance was measured at 544 nm using a Hidex Chameleon Multilabel Microplate Reader. All analyses were performed in triplicate.

### Tricine–Sodium Dodecyl Sulfate–Polyacrylamide Gel Electrophoresis

Proteins obtained from the purification steps were separated by tricine–sodium dodecyl sulfate–polyacrylamide gel electrophoresis (tricine–SDS–PAGE), adapted from the method described by Schägger and Von Jagow.^36^ An ultralow molecular weight protein marker (1060–26 600 Da; Sigma-Aldrich) was used to estimate apparent molecular masses. A volume of 5 μL of each sample was loaded per well. After electrophoresis, gels were stained with 0.025% Coomassie Blue G in distilled water containing 10% acetic acid for approximately 1 h under gentle agitation and subsequently destained with 10% acetic acid until protein bands were clearly visible.

### Immunodetection by Western Blotting

Following tricine–SDS–PAGE, proteins were transferred onto nitrocellulose membranes at a constant current of 1 mA/cm^2^ for 2 h. Transfer efficiency was verified by rapid staining with 0.1% Ponceau S. Membranes were washed with ultrapure water and blocked for 1 h in PBS (10 mM NaH_2_PO_4_, 3 mM KCl, 1.5 mM KH_2_PO_4_, 140 mM NaCl) containing 2% skim milk (Molico) and 0.05% Tween 20 (v/v).

Membranes were incubated overnight (16 h) at 4 °C with a primary anti-LTP pepper antibody (1:2000 in PBS containing skim milk, without Tween 20). After 10 washes of 5 min each with PBS, membranes were incubated for 2 h at room temperature with a peroxidase-conjugated antirabbit IgG secondary antibody (1:2000). The antibody was produced as previously described by Diz et al.^16^

Immunoreactive bands were developed in 40 mM Tris–HCl buffer (pH 7.5) containing 1 mg/mL DAB, 100 mM imidazole, and 0.03% hydrogen peroxide. The reaction was stopped with distilled water when bands became visible.

### Acquisition and Processing of Mass Spectrometry Data

Protein bands obtained by SDS–PAGE were excised using a sterile scalpel and cut into approximately 1 mm^3^ cubes. Each gel piece was transferred to a 1.5 mL microtube containing 1 mL of destaining solution composed of 50 mM ammonium bicarbonate (AmBic) and acetonitrile (ACN) at a 1:1 (v/v) ratio and incubated overnight at room temperature under gentle agitation. The solution was replaced with 200 μL of fresh destaining solution and incubated for 1 h, after which it was removed. Gel pieces were dehydrated with 500 μL of 100% ACN for 1 min, and this step was repeated once.

For reduction, 200 μL of 10 mM dithiothreitol (DTT) prepared in 100 mM AmBic was added, and samples were incubated at 55 °C for 30 min under gentle agitation. After removal of the solution, gel pieces were dehydrated with ACN and alkylated with 200 μL of 55 mM iodoacetamide (IAA; Merck) in 100 mM AmBic, followed by incubation for 30 min at room temperature in the dark. For enzymatic digestion, 200 μL of cold trypsin solution (enzyme/protein ratio of 1:100) prepared in 10 mM AmBic containing 10% ACN (V5111, Promega) was added. Samples were kept at 4 °C for 30 min and subsequently incubated at 37 °C for 18 h.

Peptides were extracted with 200 μL of 5% formic acid in 100% ACN (1:2, v/v) at 37 °C for 30 min, dried in a SpeedVac concentrator, and resuspended in 50 μL of 0.1% formic acid in 50 mM AmBic for mass spectrometry analysis.

Peptide analysis was performed using a NanoAcquity UPLC system coupled to a Synapt G2-Si HDMS mass spectrometer (Waters, Manchester, UK). Two micrograms of peptides were injected per run. Samples were initially loaded onto a C18 trap column (180 μm × 20 mm, 5 μm; Waters) at a flow rate of 5 μL/min for 3 min and subsequently separated on a C18 nanoAcquity HSS T3 column (75 μm × 150 mm, 1.8 μm) at 400 nL/min and 45 °C. The mobile phases consisted of solvent A (water containing 0.1% formic acid) and solvent B (acetonitrile containing 0.1% formic acid). The gradient of solvent B increased from 7% to 40% over 92.72 min, reached 99.9% at 106 min, returned to 7% at 106.1 min, and was maintained until 120 min.

Data acquisition was carried out in high-resolution positive-ion mode (HCD MS/MS) with ion mobility and data-independent acquisition. The ion-mobility wave speed was set to 600 m·s^–1^, and the transfer collision energy ranged from 19 to 55 V (high-energy mode). The source temperature was maintained at 70 °C, with cone and capillary voltages of 30 V and 2750 V, respectively. Time-of-flight (TOF) scans were acquired every 0.5 s in the *m*/*z* range of 50–2000. Human-[Glu^1^]-fibrinopeptide B (Sigma-Aldrich; 100 fmol/μL) was used as an external calibrant, with lock mass correction applied every 30 s. Data were acquired and processed using ProteinLynx Global Server software (PLGS v.3.0.2, Waters). Peptide identification was performed using the *C. chinense* reference proteome (UP000224522).

### Prediction and Visualization of Three-Dimensional Protein Structures

Three-dimensional protein structures were retrieved from the AlphaFold Protein Structure Database using the corresponding FASTA sequences. The predicted models were visualized, analyzed, and edited using UCSF Chimera to display peptide chains and to highlight regions identified by the proteomic mapping.

### Sample Preparation for Lipid Membrane Analysis and Protein Interactions

The following lipids were purchased from Avanti Polar Lipids, Inc. (Alabaster, AL) and used without further purification: 1-palmitoyl-2-oleoyl-*sn*-glycero-3-phosphocholine (POPC), 1-palmitoyl-2-oleoyl-*sn*-glycero-3-phospho- *l* -serine (POPS), hen egg sphingomyelin (SM), and ergosterol (Erg). Small unilamellar vesicles (SUVs) were prepared using a modified version of the method described by Crusca et al.^37^

Briefly, stock solutions of phospholipids dissolved in chloroform were mixed in glass tubes at the desired molar ratios. The organic solvent was evaporated under a gentle nitrogen stream to form a thin lipid film, which was subsequently vacuum-dried for at least 2 h to ensure complete solvent removal. The lipid film was hydrated at 20 °C with ultrapure water and subjected to six alternating freeze–thaw cycles to promote homogeneous dispersion. The resulting multilamellar vesicle suspension was sonicated for 2 min using a Vibra-Cell VCX 500 tip sonicator (Sonics & Materials, Inc.), with 10 s on and 20 s off cycles at 25% amplitude to generate SUVs. Following sonication, samples were centrifuged at 13,000 rpm for 10 min in an Eppendorf 5424R benchtop centrifuge to remove insoluble aggregates and potential titanium particles released from the sonicator tip.

The following lipid compositions were used to mimic different biological membranes: (i) POPC/POPS (50/50 mol %) to represent a sterol-free fungal membrane; (ii) POPC/POPS/Erg (35/35/30 mol %) to model an ergosterol-containing fungal membrane; and (iii) POPC/SM (55/45 mol %) to simulate a mammalian plasma membrane. Protein stock solutions were prepared in ultrapure water and mixed with the SUVs immediately before analysis to achieve the desired protein-to-lipid molar ratios. Protein concentration was determined by measuring absorbance at 280 nm using a NanoDrop One spectrophotometer (Thermo Fisher Scientific, Inc.). Liposome preparations were independently repeated to ensure reproducibility of the experiments.

### Circular Dichroism Analysis

Circular dichroism (CD) spectra were recorded at 20, 37, and 90 °C using a Jasco J-815 spectropolarimeter equipped with a Jasco Peltier PTC-3515 temperature controller. Measurements were performed in a 1 mm path length quartz cuvette, using a scan speed of 50 nm·s^–1^, a wavelength range of 350–190 nm, a data pitch of 1 nm, and a bandwidth of 2 nm. Thermal unfolding was monitored at 222 nm over a temperature range from 20 to 90 °C with a heating rate of 1 °C·min^–1^.

Protein samples were prepared at a concentration of 30 μM in ultrapure water and analyzed in the absence and presence of SUVs with different lipid compositions. Lipid concentration was fixed at 1 mM to minimize light scattering and absorption-flattening effects, maintaining the photomultiplier tube (PMT) voltage below 600 V. CD spectra were processed using CDToolX, averaging the six best scans for each measurement and subtracting the corresponding SUV spectra as baseline.^38^ All CD measurements were performed in triplicate using independently prepared samples, and representative spectra are shown.

### Inhibition Assay of the Activities of Different α-Amylases

Inhibitory activity against α-amylases was determined by measuring the residual hydrolytic activity of human salivary, porcine pancreatic, and *T. molitor* enzymes, with adaptations from Silva et al. and Resende et al.^31,39^ Seed fractions from *C. chinense* (P10, R1, R2, and R3) were prepared at a concentration of 50 μg·mL^–1^ and incubated at 37 °C for 30 min with 1 μL of α-amylase (human salivary, porcine pancreatic, or *T. molitor*).

The enzyme volume was selected based on a previously established activity curve corresponding to 10 U of α-amylase, combined with 25 μL of 1% (w/v) starch solution (Sigma-Aldrich), in a final reaction volume of 100 μL. After incubation, 200 μL of dinitrosalicylic acid (DNS) reagent (4.5% sodium hydroxide, 3,5-dinitrosalicylic acid, 1% sodium double tartrate, 45 g potassium sodium tartrate, and 2 g crystalline phenol) was added. Reaction mixtures were heated at 100 °C for 5 min, cooled to room temperature, and the absorbance was measured at 540 nm using a Spectroquant Pharo 100 spectrophotometer (Merck) to quantify substrate hydrolysis.

All assays were performed in triplicate, and results are expressed as mean ± standard deviation. One unit of α-amylase activity was defined as a change of 0.1 in absorbance at 540 nm over 30 min.^32^ Percentage inhibition was calculated relative to a positive control containing 5 mM EDTA (considered 100% inhibition). Bovine serum albumin (BSA) at 10 μg·mL^–1^ was used as the negative control.

### Protein Structure Analysis

The α-amylase structural model was obtained from the Protein Data Bank (https://www.rcsb.org/; code 1JAE) for a larval enzyme from *T. molitor*. LTP three-dimensional structures were modeled in SWISS-MODEL (https://swissmodel.expasy.org/) using FASTA sequences PHU29557.1 and PHU29554.1 (GenBank: https://www.ncbi.nlm.nih.gov/genbank/). PyMOL (https://pymol.org/) was used to highlight regions of interest.

### LTP Docking with α-Amylase (Molecular Docking)

Docking between LTPs and α-amylase was performed using ClusPro V2 (https://cluspro.bu.edu/),^40^ HADDOCK (https://haddock.science.uu.nl/services/HADDOCK2.4/),^41^ and HDock (http://hdock.phys.hust.edu.cn/).^42^ Complexes were ranked with PRODIGY (https://bianca.science.uu.nl/prodigy/).^43^ The best LTP-α-amylase complex was selected by the lowest free energy (Δ*G*). Interface interactions were analyzed using LigPlot+ (https://www.ebi.ac.uk/thornton-srv/software/LigPlus/).^44^

### Assessment of Hemolytic Activity

Hemolytic activity of the fractions was assessed using defibrinated sheep erythrocytes (sRBCs) as described by Oren and Shai, with adaptations.^45^ Fresh EDTA-treated red blood cells were washed three times with 0.15 M NaCl by centrifugation at 2400*g* for 10 min and resuspended to 1% (v/v) in the same solution. Peptide fractions were diluted in saline to 35.5, 75, 150, and 300 μg/mL. For each assay, 50 μL of dilution was incubated with 50 μL of sRBC suspension at 37 °C for 1 h. Samples were centrifuged at 2400*g* for 10 min, and 100 μL of supernatant was transferred to a 96-well plate. Hemoglobin release was measured at 405 nm. Positive control: 1% Triton X-100. Negative control: RBCs incubated with PBS. The percentage of hemolysis was calculated as % hemolysis = ((A_sample – A_neg)/(A_pos – A_neg)) × 100. All assays were performed in triplicate based on four independent experiments. Data were analyzed by ANOVA followed by Tukey’s test (*p* < 0.05) and are presented as mean ± SD.

## Data Availability

All the data generated or analyzed during this study are included in this published article.
